# Paroxysmal Dystonia: An Etiology Not to Be Missed in an Older Adult Patient

**DOI:** 10.1002/mdc3.70407

**Published:** 2025-10-23

**Authors:** Valentin Mira, Jan Patrick Stellman, Stephan Grimaldi

**Affiliations:** ^1^ Service de Pathologie du Mouvement Assistance publique—Hôpitaux de Marseille Marseille France; ^2^ Aix‐Marseille University, CEMEREM, CNRS, CRMBM, UMR 7339; APHM La Timone Service de Neuroradiologie Marseille France

**Keywords:** paroxysmal kinesigenic movement, thalamus, movement disorder

## Question

A 57‐year‐old man with a history of hypertension, managed with Ramipril, exhibited paroxysmal and painful contractures on the right‐side hemibody for 10 days. The contractures initially involved his right hand and subsequently affected the whole hemibody (Video 1). Episodes were triggered by sudden movements, lasted less than a minute, and recurred every 5 minutes. He reported no similar episode in the past. One month prior, he experienced a brief and transient right‐sided weakness; he did not seek medical attention at that time. Neurological examination conducted between episodes was unremarkable. Brain tomography, electroencephalogram, and laboratory tests revealed no abnormalities.

**VIDEO 1:** Example of the patient's paroxysmal dystonia episode. In the first part of the video, a shadow on the affected leg creates a superficial rippling appearance; this is a visual artifact, not true muscle hyperexcitability disorder.

## What Is Your Main Diagnostic Hypothesis?

1. Primary paroxysmal kinesigenic dyskinesia (PKD)
2. Paroxysmal exercise‐induced dystonia
3. Secondary paroxysmal kinesigenic dystonia
4. Paroxysmal non‐kinesigenic dyskinesia

## Answer

The answer is 3: Secondary paroxysmal kinesigenic dystonia.

**FIG. 1:**
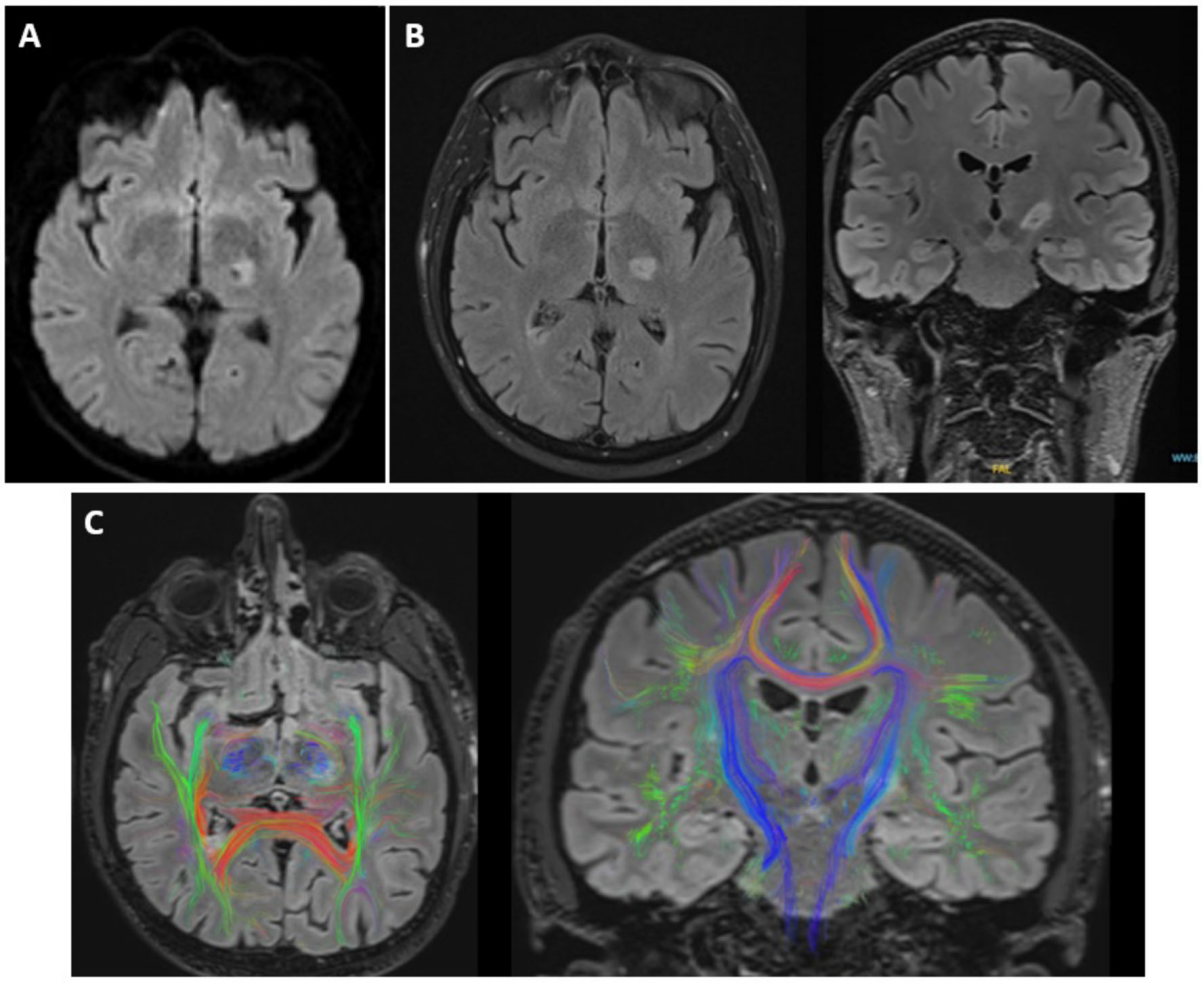
Brain MRI with diffusion‐weighted imaging (DWI) (**A**) and fluid‐attenuated inversion recovery (FLAIR) (**B**) shows a recent ischemic lesion in the left internal capsule and thalamus. Fiber tractography (**C**) reveals impairment of the pyramidal tract (in blue).

Because episodes are triggered by sudden movements, a kinesigenic origin is suspected. Late onset is a strong argument for a secondary cause, as genetic PKD generally appears in the second decade.^1^ Painfulness is another significant red flag for primary PKD.^1^ A history of neurological deficit strongly suggests the need for further investigations to find a lesional cause. Here, brain MRI revealed a DWI and FLAIR hypersignal within the internal capsule and left thalamus, specifically affecting the ventral lateral and ventral anterior nuclei, consistent with a recent ischemic event (Fig. 1A,B). Fiber tractography demonstrated impairment of the pyramidal tract (Fig. 1C). The patient's clinical course supported the diagnosis of paroxysmal dystonia secondary to a lacunar infarction, as post‐stroke dystonia classically manifests with delayed onset after injury.^2^ This case highlights the role of thalamic dysfunction in the development of symptoms by disinhibiting thalamocortical pathways and increasing cortical excitability.^1^, ^2^ As recommended in PKD, a low dose of carbamazepine (100 mg, off‐label) was introduced and led to the resolution of the patient's symptoms.^1^

## Author Roles

(1) Research project: A. Conception, B. Organization, C. Execution; (2) Statistical analysis: A. Design, B. Execution, C. Review and critique; (3) Manuscript preparation: A. Writing of the first draft, B. Review and critique.

V.M.: 1A, 3A.

J.P.S.: 1A, 3B.

S.G.: 1B, 3B.

## Disclosures

**Ethical compliance statement:** Given the anonymous nature of the data presented, institutional review board or ethics committee approval was not required for this case report. We have obtained written consent from the patient for this manuscript. We confirm that we have read the journal's position on issues involved in ethical publication and affirm that this work is consistent with those guidelines.

**Funding Sources and Conflict of Interest:** No specific funding was received for this work. The authors declare that there are no conflicts of interest relevant to this work.

**Financial disclosures for the previous 12 months:** The authors declare that there are no additional disclosures to report.

## Data Availability

Data sharing not applicable to this article as no datasets were generated or analysed during the current study.
